# Regional Pericarditis Mimicking Inferior Myocardial Infarction following Abdominal Surgery

**DOI:** 10.1155/2014/301976

**Published:** 2014-03-05

**Authors:** Ahmad T. Alhammouri, Bassam A. Omar

**Affiliations:** Division of Cardiology, University of South Alabama, Mobile, AL 36617, USA

## Abstract

Acute pericarditis is common but illusive, often mimicking acute coronary syndrome in its clinical and electrocardiographic presentation. Regional pericarditis, though rare, presents further challenge with a paucity of published diagnostic criteria. We present a case of postoperative regional pericarditis and discuss helpful electrocardiographic findings. A 66-year-old male with history of open drainage of a liver abscess presented with abdominal pain and tenderness. CT of the abdomen was concerning for pneumatosis intestinalis of the distal descending colon. He underwent lysis of liver adhesions; exploration revealed only severe colonic impaction, for which he had manual disimpaction and peritoneal irrigation. Postoperatively, he developed sharp chest pain. Electrocardiogram revealed inferior ST elevation. Echocardiogram revealed normal left and right ventricular dimensions and systolic function without wall motion abnormalities. Emergent coronary angiography did not identify a culprit lesion, and left ventriculogram showed normal systolic function without wall motion abnormalities. He received no intervention, and the diagnosis of regional pericarditis was entertained. His cardiac troponin was 0.04 ng/dL and remained unchanged, with resolution of the ECG abnormalities in the following morning. Review of his preangiography ECG revealed PR depression, downsloping baseline between QRS complexes, and reciprocal changes in the anterior leads, suggestive of regional pericarditis.

## 1. Introduction

Prompt evaluation of an electrocardiogram (ECG) remains the basis for the initial workup and early implementation of life-saving therapy in patients presenting with chest pain suggestive of acute coronary syndrome (ACS). Vigilance, however, is recommended in the interpretation of the ECG, paying attention to atypical clinical or electrocardiographic features which may point at an alternative diagnosis, mandating further diagnostic studies and different management. We present a case of regional pericarditis with electrocardiographic features mimicking an inferior myocardial infarction, prompting early cardiac catheterization. Subtle ECG findings, however, can serve as clues to the presence of pericarditis rather than myocardial ischemia. Review of pertinent literature is provided.

## 2. Case Report

**Figure 1:**
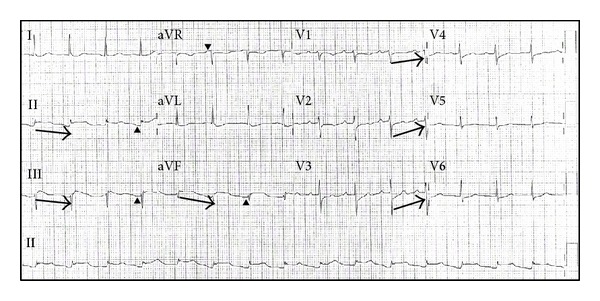
Initial electrocardiogram, showing inferior ST-segment elevations in II, III, and aVF with reciprocal ST-segment depressions in the anterior leads (V1–V4). Arrowheads show PR depression in the inferior leads, with corresponding PR elevation in aVR. Arrows show the downsloping ECG baseline from one QRS complex to the following PR-segment (Spodick's sign) in the inferior leads, with corresponding upsloping ST-segment depression in the anterior leads (“reverse” Spodick's sign).

**Figure 2:**
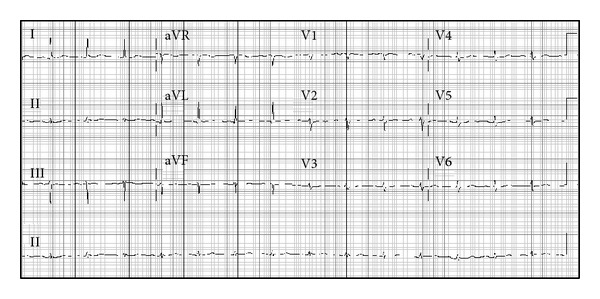
Repeat electrocardiogram on the following morning showing return of the ST-segments to baseline.

A 66-year-old white male with known hypertension and diabetes mellitus and prior open drainage of a liver abscess presented to our emergency department with abdominal pain and tenderness. The patient underwent computed tomography (CT) scan of the abdomen which revealed findings consistent with fecal impaction and pneumatosis intestinalis of the distal descending colon. He was taken to the operating room where he underwent extensive lysis of liver adhesions thought to have been caused by his prior liver surgery, and exploration revealed only severe colonic impaction. He underwent manual disimpaction of fecal material and gas, peritoneal irrigation, and closure. His initial postoperative course was uneventful, and he was quickly weaned off the ventilator. However, later in the evening he developed chest pain, described as retrosternal sharp to tight feeling, without radiation. Deep inspiration caused worsening of his abdominal pain at the incision site but did not alter his chest pain severity. The patient's blood pressure and heart rate remained stable. ECG revealed inferior ST elevations with reciprocal ST depressions in the anterior leads suspicious of myocardial injury (Figure 1); therefore, the decision was to call in the cardiac catheterization laboratory team for urgent angiography. An echocardiogram, performed while awaiting angiography, revealed normal left and right ventricular dimensions and systolic function without obvious wall motion abnormalities or pericardial effusion, with a pulmonary artery systolic pressure estimated at 34 mmHg. Coronary angiography revealed normal right coronary artery, with moderately severe mid left anterior descending artery lesion, which was not deemed to be a culprit, since his left ventriculogram showed normal left ventricular systolic function without wall motion abnormalities. He received no intervention, and the diagnosis of regional pericarditis was entertained. Three sets of cardiac troponin I (cTnI) were less than 0.04 ng/mL. His postoperative pain management was continued, with resolution of the ECG abnormalities, the following morning (Figure 2).

## 3. Discussion

Barnes and Burchell [1] reported fourteen cases of acute pericarditis, diagnosed on clinical and electrocardiographic grounds, with ECG changes which simulated acute coronary occlusion. Later reports confirmed the often difficult differentiation between acute pericarditis and coronary occlusion [2, 3]. The problem appears to be further confounded when pericarditis is regional, with electrocardiographic features nearly indistinguishable from localized myocardial infarction, which may lead to erroneous treatment [4]. Millaire et al. [5] reported 19% incidence of thrombolysis in patients with myopericarditis erroneously diagnosed as myocardial infarction; although no severe cardiac or pericardial complications or arrhythmia were reported, there was one case of noncompressive pericardial effusion. Salisbury et al. [6] reported that coronary angiography was performed within 24 hours of presentation in 25% of patient with final diagnosis of acute pericarditis and ST elevation; it is of note that 5% received thrombolytics before transfer to the tertiary facility. Barge-Caballero et al. [7] reported that the prevalence of catheterization laboratory false alarm (no culprit lesion identified) is as 7.2% in a regional primary angioplasty network; 8% of these were eventually diagnosed as pericarditis.

Characteristic ST and T-wave abnormalities and evolution patterns have long been observed in patients with postinfarction regional pericarditis [8]. Localized electrocardiographic ST-T abnormalities corresponding to the area of infarction were also reported experimentally in the postinfarction pericarditis dog model [9]. Oliva et al. [10] reported atypical pattern of T-wave evolution in all patients clinically identified to have postinfarction pericarditis; either persistently positive T-waves or reversal of initially inverted T-waves after 48 hours existed in every patient irrespective of infarction. The sensitivity and specificity of these T-wave alterations were reported to be 100% and 77%, respectively [11]. Dorfman and Aqel [12] reviewed the pericardial manifestation of acute myocardial infarction. They concluded that regional pericarditis following myocardial infarction should be considered in patients with recurrent chest pain in the setting of atypical T-wave evolution and persistent ST-segment elevation, so that anticoagulation or thrombolysis may be avoided in favor of early angiography to exclude reinfarction.

Regional pericarditis has also been observed in the absence of infarction. Youssef et al. [13] reported a 49-year-old man with abdominal free air one month after kidney-pancreas transplant, who was noted to have inferior ST elevation on a preoperative ECG. The patient denied chest pain; cardiac enzymes did not indicate myocardial infarction and an echocardiogram revealed no wall motion abnormalities. Upon further analysis of the ECG, the ST-segment elevation was noted to be concave with upright T-waves, PR depression, and reciprocal changes in aVR and V1, all suggestive of regional pericarditis. Jain [14] reported a 92-year-old man with generalized malaise; his ECG revealed anterior ST elevation consistent with acute MI. Due to the absence of chest discomfort, an echocardiogram was performed which revealed mild generalized hypokinesis. Subsequent ECGs showed evolutionary changes of pericarditis. Pyxaras et al. [15] reported a 59-year-old male with pleural mesothelioma who presented with chest pain and localized ST elevation. Coronary angiography excluded an acute coronary occlusion, whereas high-resolution CT scan of the chest revealed tumor infiltration and associated acute inflammation of the pericardial sac.

The American Heart Association, American College of Cardiology Foundation, and Heart Rhythm Society (AHA/ACCF/HRS) guidelines [16] recommend that the ECG report should include a qualitative description of ST-segment abnormalities with due consideration for the age and gender of the patient. Possible causes, depending on other ECG abnormalities and knowledge of any pertinent clinical information, also may be included. Factors, other than acute ischemia, that may cause ST-segment elevation include but are not limited to pericarditis, elevated serum potassium, Osborn waves, acute myocarditis, certain cardiac tumors, and the normal variant referred to as “early repolarization” [17]. Wang et al. [18] reviewed ST-segment elevation in conditions other than acute myocardial infarction; they proposed that the shape of the ST-segment elevation, the leads involved, the clinical setting in which the elevation occurs, and awareness of the conditions that mimic infarction are all crucial in identifying noninfarction etiologies for the ST-segment elevation.

Although the absence of elevated cTnI may help differentiate pericarditis from myocardial infarction, cTnI elevation is often observed in viral or idiopathic acute pericarditis [19]. Echocardiography can be very useful in excluding regional wall motion abnormalities and identifying pericardial effusion, especially in atypical presentations of pericarditis [20]. In the acute setting, however, prompt differentiation of pericarditis from myocardial injury by ECG remains of paramount importance to avoid delay in reperfusion while awaiting the results of cardiac biomarkers or echocardiography. Spodick [21] described PR-segment depression, consistent with the subepicardial atrial injury of acute pericarditis, in 82% of 50 patients with unequivocal pericarditis. The same author [22] stressed the importance of considering the TP-segment to be the electrocardiographic baseline, to avoid mistaking PR-segment depression for ST-segment elevation. The combination of PR-segment depression and a downsloping ECG baseline from one QRS complex to the following PR-segment, previously termed “Spodick's sign” [23], serves as a helpful feature in differentiating acute pericarditis from acute myocardial injury.

Our patient developed early postoperative inferior ST elevations, together with reciprocal anterior ST depressions, suggestive of an acute inferior ST-elevation myocardial infarction (Figure 1), which prompted cardiac catheterization. Coronary angiography excluded any culprit lesion which could explain the ST-segment elevations. However, upon closer analysis of the ECG, the ST-segment elevations in the inferior leads exhibit a downsloping baseline from one QRS complex to the following PR-segment, characteristic of the previously described Spodick's sign. The anterior leads interestingly show a “reciprocal” upsloping baseline from one QRS complex to the following PR-segment, suggestive of “reverse” Spodick's sign. Complete resolution of the patient's ECG abnormalities (Figure 2), the absence of wall motion abnormalities, and the lack of elevation of cTnI all support the diagnosis of regional pericarditis.

## 4. Conclusion

Pericarditis is a common condition with clinical and electrocardiographic features which can mimic ACS. The subtle differences between the two conditions are often overlooked due to the fear of missing the more serious diagnosis of ACS and the window for timely reperfusion. Even though ancillary tests, such as echocardiography and cardiac biomarkers, can aid in diagnosing pericarditis or excluding ACS, careful attention to specific characteristic findings on the ECG remains the mainstay of initial and timely recognition and triage of patients to avoid the potentially harmful consequences of inappropriate therapy. Helpful electrocardiographic features in differentiating acute pericarditis from ACS include PR-segment depression, a downsloping ECG baseline from one QRS complex to the following PR-segment (Spodick's sign), and, in regional pericarditis, reciprocal ST changes (“reverse” Spodick's sign).
